# Bullying Victimization and Problem Video Gaming: The Mediating Role of Externalizing and Internalizing Problems

**DOI:** 10.3390/ijerph18041930

**Published:** 2021-02-17

**Authors:** Jérémie Richard, Loredana Marchica, William Ivoska, Jeffrey Derevensky

**Affiliations:** 1 International Centre for Youth Gambling Problems and High-Risk Behaviors, Department of Educational and Counselling Psychology, McGill University, Montreal, QC H3A 1Y2, Canada; loredana.marchica@muhc.mcgill.ca (L.M.); jeffrey.derevensky@mcgill.ca (J.D.); 2 Department of Psychology, The Montreal Children’s Hospital, Montreal, QC H4A 3J1, Canada; 3 Alcohol, Drug Addiction and Mental Health Services Board of Wood County Ohio, Bowling Green, OH 43402, USA; billivoska@gmail.com

**Keywords:** bullying victimization, cyberbullying, externalizing problems, gaming disorder, internalizing problems, problem video gaming

## Abstract

Background: Adolescent victims of bullying are more likely to experience a range of mental health problems. Although research has investigated the relationship between bullying victimization and various addictive behaviors, the impact of bullying on problem video gaming (PVG) remains largely unexplored. The purpose of this study is to investigate the relationship between bullying victimization and PVG as mediated by the presence of internalizing and externalizing problems. Methods: Survey responses were collected from 6353 high-school students aged 12 to 18. Measures include bullying victimization (physical, verbal, cyber and indirect), internalizing (e.g., anxious and depressive symptoms) and externalizing (e.g., aggressive and delinquent problems) problems, and PVG (measured by the Internet Gaming Disorder Scale–Short Form). Results: Mediation analyses indicated that the relationship between verbal bullying and PVG was completely mediated by the presence of internalizing and externalizing problems. The relationship between physical bullying and PVG was completely mediated by externalizing problems and the relationship between cyberbullying and PVG was completely mediated by internalizing problems. Lastly, the relationship between indirect bullying and PVG was partially mediated by externalizing and internalizing problems. Conclusions: Results suggest that different types of bullying victimization are differentially associated with PVG, with mental health symptoms significantly mediating this relationship.

## 1. Introduction

Technological developments in recent decades have increased engagement with screen-based technologies including computers/laptops, video gaming consoles, smartphones and tablets among children and adolescents. Recent reports have indicated that nearly 3.1 billion people worldwide play video games [1], with over 70% of youth reporting having played video games over the past year [2,3,4,5,6]. With gaming activities representing an increasingly important part of the leisure and social pursuits of youth, concerns have been raised relevant to the potential for problematic engagement in gaming [7]. In 2019, the World Health Organization (WHO) recognized gaming disorder (GD) as a mental disorder characterized by a persistent engagement in gaming, impaired control over gaming, and functional impairment due to gaming over a period of at least 12 months [8]. The inclusion of this disorder in the ICD-11 was not without controversy, with the DSM-5 identifying internet gaming disorder as a disorder requiring further research before official inclusion [9], and some experts claiming that labeling problematic engagement in gaming as a disorder would be premature [10,11]. Nevertheless, reviews of GD prevalence have broadly established that prevalence rates of GD range from 0.7% to 27.5% globally [12,13,14], with results from a recent meta-analysis indicating a pooled prevalence rate of 1.96% to 3.05% depending on the sampling criteria and measurement tool [15]. Specific to GD, adolescents and males in particular have been found to endorse higher rates of GD compared to adults and females [15].

GD, or more broadly, problem video gaming (PVG; subclinical levels of GD), has been associated with a number of psychosocial consequences across the lifespan [16]. In childhood and adolescence, GD has been found to increase levels of anxiety, depression, aggression, emotional distress and loneliness, while negatively impacting youth’s self-esteem, life satisfaction, school performance and parental relationships [17,18,19,20,21,22]. Moreover, several risk factors for GD in childhood and adolescence have also been identified, including emotion dysregulation, aggression, impulsivity, attention difficulties, peer and parental problems, and peer victimization [18,19,22,23,24,25]. Although PVG has been associated with increases in time spent playing video games, not all youth that report frequent video game playing report symptoms of PVG [26,27]. Overall, PVG appears to be closely associated with a number of mental health and social outcomes with the potential for reciprocal relationships between these factors. 

The most common form of peer difficulties reported by youth is bullying victimization, affecting 28% to 36% of children and adolescents over the past year [28,29]. Bullying is typically defined as repetitive aggressive acts made with a harmful intent, where there is an imbalance of power between the aggressor and the victim [30,31]. Studies have identified that adolescent victims of bullying report higher rates of internalizing [32,33,34,35] and externalizing problems [36,37,38,39], while also being associated with physical health problems, academic and familial difficulties [40,41,42].

A recent area of investigation has been the relationship between bullying victimization, video game playing and PVG. A number of studies have identified associations between increased time playing video games and school/cyberbullying victimization among youth [43,44,45,46,47]. These studies have hypothesized that video game playing can be used as a form of emotion regulation and escape for some youth that experience more frequent bullying victimization. Additionally, some studies have found that frequent video game playing has been associated with increases in cyberbullying perpetration (or of being both a victim and perpetrator of bullying [i.e., bully-victims]), within and outside online gaming environments [43,44,45,48,49,50,51]. Specific to PVG, direct positive relationships have been identified between bullying victimization and PVG [52,53,54]. However, little research has investigated the potential mediators explaining the relationship between these variables. One study, by Zhao and colleagues [55], identified that a lack of meaning in life mediated the relationship between bullying victimization and PVG among girls, and in boys, the presence of meaning in life moderated this relationship (i.e., meaning buffered against increases in PVG). 

Overall, although research has identified significant associations between bullying victimization, mental health symptoms, and PVG, no studies have presented these variables in an integrated model to investigate their sequential associations. Of note, it is possible that the relationship between bullying victimization and PVG is mediated by the presence of mental health symptoms, as externalizing and internalizing problems have been found to fully mediate the relationship between bullying victimization and a number of other addictive behaviors including alcohol, cannabis, cigarette use, and gambling [56]. With regards to PVG, it is possible that similar relationships are present due to the etiological, clinical, and phenomenological similarities between substance and non-substance addictions [57,58,59]. Moreover, there is a need for mediation models to account for the frequency of engagement in gaming as the latter has been independently associated with increases in both bullying victimization and PVG symptoms [27,43,46,47]. Finally, as a majority of studies have looked at either cyberbullying or school bullying in general, it is crucial to differentiate between different types of bullying victimization (e.g., verbal, physical, cyber, indirect [e.g., spreading mean rumors or being kept out of a “group”]), as they have been found to be differentially associated with externalizing (larger effects with physical bullying) and internalizing (larger effects with indirect, cyber and verbal bullying) problems [60,61,62,63].

Given the potential associations between bullying victimization, mental health symptoms and PVG, the present study aims to analyze the relationships between different types of bullying victimization and PVG severity, as mediated by externalizing and internalizing problems. It was hypothesized that a higher frequency of any type of bullying victimization would be associated with greater externalizing and internalizing problems. Second, it was hypothesized that externalizing and internalizing problems would mediate the relationship between bullying victimization and PVG. Third, as gaming frequency appears to be closely related to PVG, it was hypothesized that the frequency of video game play would in turn mediate the relationship between externalizing and internalizing problems and PVG. 

## 2. Materials and Methods

### 2.1. Participants and Procedure

The current study employed data from the 2019 Alcohol, Drug Addiction and Mental Health Services (ADAMHS) Board/Wood County Educational Service Center Survey on Alcohol and Other Drug Use among adolescents in Wood County, Ohio. Ethical approval was granted by the ADAMHS ethics committee. Students completed an anonymous paper-and-pencil survey administered by their classroom teacher. In each school, trained addiction counsellors coordinated the survey distribution and assisted teachers with administration when required. Participants were informed that their survey responses were confidential and that they could withdraw from participation at any time without consequence. School principals provided parents with an informed consent form to ensure parental assent for participation. Information regarding the survey was provided to all parents by letter and were available for viewing on each school district website. Parents could elect for their children to opt out by informing their school principal.

Of a total of 7573 students from grades 7 through 12 who participated in this study, 763 were excluded due to suspected insincere responding (i.e., reporting engagement in all gambling activities at all times and providing inconsistent responses). Of the remaining 6810 adolescents, 57 were removed for not meeting age criteria (i.e., ages 12–18), 360 were removed for omitting their gender, and 40 were removed for having greater than 50% missing items on either the internalizing or externalizing problem factors of the Problem Severity Scale. A total of 6353 adolescents (M_age_ = 14.74 years, SD = 1.76, 50.4% male) were included in the final sample.

### 2.2. Measures

#### 2.2.1. Demographic Characteristics

All participants indicated their gender, age, grade level, and ethnicity (e.g., White, Hispanic, Black) at the beginning of the survey.

#### 2.2.2. Bullying Victimization

Bullying experiences were defined as purposeful acts by which bullies use their power to threaten, harass, or hurt others. Participants were asked to report how frequently they had been bullied over the past month on a 5-point Likert scale (0 = “Not at all”; 1 = “Once or twice”; 2 = “Several times”; 3 = “Often”; 4 = “Most of the time”). Four types of bullying experiences were explored: *physical*, *verbal*, *cyber*, and *indirect* bullying.

#### 2.2.3. Externalizing and Internalizing Problems

The Ohio Scales Youth Problem Severity Scale (PSS) is a 20-item self-report measure that assesses common mental health symptoms and problem behaviors over the past 30 days based on common presenting problems by youth with emotional disturbances and problem behaviors based on the DSM-IV [64]. Scores were calculated by summing the participants’ ratings of each item on a 6-point scale (0 = “Not at all”; 1 = “Once or twice”; 2 = “Several times”; 3 = “Often”; 4 = “Most of the time”; and 5 = “All of the time”. Psychometric studies have identified the presence of an overarching two-factor structure of the PSS including an internalizing and externalizing problems factor [65]. Scores for internalizing problems (nine items) ranged from 0 to 45 (*M* = 7.48; *SD* = 10.12), while scores for externalizing problems (eleven items) ranged from 0 to 55 (*M* = 7.26; *SD* = 7.94). Within the present sample, the internal consistency for the PSS was excellent (Cronbach α = 0.93).

#### 2.2.4. Gaming Frequency

Participants were asked to report how often they spent at least two hours daily playing video games online or offline over the previous year. Participants indicated responses on a 5-point Likert scale (0 = “Not at all”; 1 = “Less than once a month”; 2 = “About once a month”; 3 = “About once a week”; 4 = “Daily”).

#### 2.2.5. Problem Video Gaming

Problem video gaming was assessed utilizing the Internet Gaming Disorder Scale-Short Form (IGDS-SF9) [66]. The IGDS-SF9 is a 9-item measurement scale adapted from the nine core criteria that define internet gaming disorder according to the DSM-5 [9]. Responses were based on a 5-point Likert scale (0 = “Never”; 1 = “Rarely”; 2 = “Sometimes”; 3 = “Often”; 4 = “Very Often”), which can be summed for a total score ranging from 0 to 45, with higher scores indicating greater severity of internet gaming disorder. A score of 35 or more was considered at risk for PVG and 146 participants scored within this range. Within the present sample, the internal consistency for the IGDS-SF9 was excellent (Cronbach α = 0.91).

### 2.3. Statistical Analysis

IBM SPSS version 25 was used to calculate the descriptive statistics and for preliminary analyses. Independent samples *t*-tests were conducted to explore gender differences in rates of bullying victimization, externalizing, and internalizing problems, the frequency of video game play, and PVG severity. Pearson’s correlation coefficients were calculated to investigate the association between the continuous variables included in the structural equation model. For the structural equation model, a parallel-serial mediation model was conducted using M*plus* version 8.0 [67]. In the parallel-serial media model, age and sex were included as covariates, and correlations were included between the four different types of bullying victimization and between externalizing and internalizing problems. Global goodness of fit for the model was assessed across several indices including the chi-square test of model fit, root mean square error of approximation (RMSEA; ≤0.05), comparative fit index (CFI; ≥0.95), and standardized root mean squared residual (SRMR; ≤0.06), with the values in brackets indicating an excellent fit [68,69]. Bootstrapping procedures were used to calculate confidence intervals for estimates of the indirect effects [70]. Confidence intervals (95% CI) from 5000 bootstrap samples are reported. Bootstrap intervals are calculated as significant when the 95% CI does not contain zero.

## 3. Results

Table 1 presents the descriptive statistics for the demographic characteristics of the sample, the frequency of bullying victimization over the past month, self-reported externalizing and internalizing problems, gaming frequency and PVG severity. Independent samples *t*-tests were utilized to investigate gender differences in the investigated variables. Results indicated that rates of physical bullying victimization were greater among males compared to females (*M* = 0.19, *SD* = 0.66 vs. *M* = 0.17, *SD* = 0.58, *t*[6340] = 7.80, *p* = 0.005). Females reported being bullied more often than males for verbal (*M* = 0.57, *SD* = 1.02 vs. *M* = 0.45, *SD* = 0.98, *t*[6344] = 25.68, *p* < 0.001), cyber (*M* = 0.29, *SD* = 0.78 vs. *M* = 0.20, *SD* = 0.70, *t*[6347] = 61.65, *p* < 0.001), and indirect (*M* = 0.50, *SD* = 1.00 vs. *M* = 0.31, *SD* = 0.87, *t*[6341] = 141.48, *p* < 0.001) bullying. No gender differences were noted for externalizing problems (*p* = 0.083). However, females reported greater internalizing problems (*M* = 9.86, *SD* = 11.21 vs. *M* = 5.11, *SD* = 8.04, *t*[5997] = 366.53, *p* < 0.001). As for the frequency of video game play, males reported more frequent participation compared to females (*M* = 2.30, *SD* = 1.71 vs. *M* = 1.44, *SD* = 1.64, *t*[6261] = 22.71 *p* < 0.001) and males also reported greater PVG severity (*M* = 14.57, *SD* = 7.21 vs. *M* = 11.59, *SD* = 5.99, *t*[6083] = 228.11, *p* < 0.001).

### 3.1. Assumption Tests for the Parallel-Serial Mediation Model

First, all included variables were measured at the interval or ratio level. Second, the absence of multicollinearity was verified through correlational analyses and was considered acceptable as the Pearson’s correlation coefficients ranged from *r* = 0.02 to *r* = 0.68 (Table 2), with no coefficients exceeding 0.90 [71]. Third, although some values of skewness and kurtosis exceeded the acceptable range (±1.00) [72], a decision was made to include these variables as the variables in question are typically positively skewed (i.e., bullying victimization, mental health symptoms, PVG severity). Third, all missing data were excluded prior to conducting the structural equation models. No significant differences were present in the demographic characteristics of the included and excluded sample.

### 3.2. Parallel-Serial Mediation Model for Bullying Victimization and Problem Video Gaming

**Figure 1:**
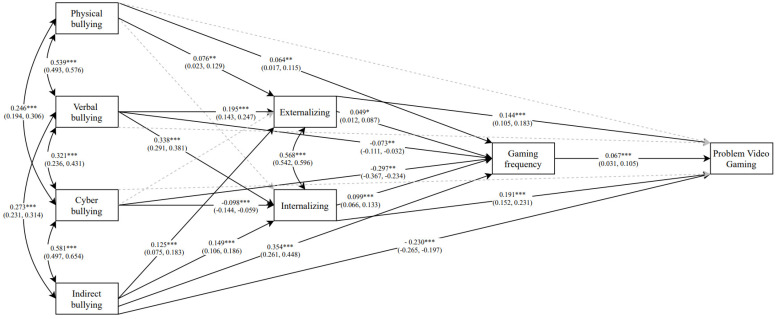
Parallel-serial mediation model whereby externalizing and internalizing problems mediate the relationship between physical, verbal, cyber and indirect bullying and gaming frequency, and gaming frequency mediating the relationship between externalizing and internalizing problems and severity of problem video gaming. All estimates are standardized with 95% confidence intervals in parentheses. Dashed lines were estimated but non-significant (*p* > 0.05). * *p* < 0.05; ** *p* < 0.01; *** *p* < 0.001.

A parallel-serial mediation model investigating the relationship between bullying victimization, externalizing and internalizing problems, gaming frequency and problem video gaming was used. There were four parallel paths by which each type of bullying victimization (i.e., physical, verbal, cyber and indirect) were hypothesized to be associated with PVG: (i) externalizing problems; (ii) internalizing problems; (iii) externalizing problems and gaming frequency; and (iv) internalizing problems and gaming frequency. The model indicated an overall acceptable fit to the data, the chi-square test was statistically significant (χ^2^ = 518.73 (8, N = 5537), *p* < 0.001), the RMSEA was 0.10, the CFI was 0.92 and the SRMR was 0.04. Path estimates are presented in Figure 1 and indirect effects in Table 3. Gender and age were included as covariates in the model.

Model results indicate that the frequencies of verbal and indirect bullying were significantly positively associated with gaming frequency and PVG severity through internalizing problems, with cyber bullying being significantly negatively associated with gaming frequency and PVG severity through internalizing problems. Moreover, the frequencies of physical, verbal and indirect bullying were significantly positively associated PVG severity through externalizing problems, with the path through gaming frequency being non-significant. Overall, the observed variables explained 16.1% of the variance in PVG severity, with complete mediation effects being noted for physical bullying through externalizing problems, verbal bullying through externalizing problems, internalizing problems and both internalizing problems and gaming frequency, and cyber bullying through internalizing problems and both internalizing problems and gaming frequency. Partial mediation effects were only noted for indirect bullying through externalizing problems, internalizing problems and both internalizing problems and gaming frequency, with the direct effect from indirect bullying to PVG severity remaining significant.

## 4. Discussion

The first objective of this study was to better understand the relationship between four types of bullying victimization often observed in high schools (verbal, physical, cyber, and indirect bullying) and PVG severity, as mediated by both externalizing and internalizing problems. Additionally, given that gaming frequency is positively correlated with PVG severity, the following study conducted a parallel-serial mediation including this variable. In line with the first hypothesis, each type of bullying was significantly and positively correlated with both externalizing and internalizing problems. This is consistent with previous research that has demonstrated that increased levels of bullying victimization are associated with various mental health symptoms [32,33,34,35,36,37,38,39,73,74].

However, some noteworthy changes in these associations were identified within the mediation model, which suggests that this study’s second hypothesis was only partially supported. Specifically, verbal and indirect bullying were positively associated with PVG through both externalizing and internalizing problems, and physical bullying was positively associated with PVG through externalizing problems. Overall, this is consistent with previous research indicating that the relationship between bullying victimization and engagement in a variety of addictive behaviors (i.e., gambling, alcohol, marijuana, and cigarette use) is significantly mediated by mental health symptoms [56]. What is unique about the present study is that this relationship also extends to gaming disorder. This not only replicates previous studies reporting positive relationships between bullying victimization and PVG [52,53,54], yet provides evidence for the role of mental health symptoms in this relationship, accounting for 16.1% of the variance in PVG severity. The extent of the variance explained in this model points to the fact that various factors other than bullying victimization, externalizing and internalizing problems may explain youth PVG, including self-esteem, impulsivity, attention difficulties, social competencies and parental relationship quality [16]. Nevertheless, along with previous research, these findings demonstrate that engagement in video gaming may be a way for individuals to cope with, escape, or avoid the uncomfortable emotions associated with being a victim of bullying [75].

Furthermore, the differences in these interrelationships based on the type of bullying victimization may be indicative of differing psychopathological pathways to PVG. For verbal and indirect bullying, although significant mediation effects were present through both externalizing and internalizing problems, the relationship appeared to be greater for internalizing problems which is consistent with past research on this subject [60,61,62,63]. These results may be indicative of possible gender differences in these associations, with females endorsing greater verbal and indirect bullying and greater internalizing problems. However, gender was added as a control variable in the mediation model to account for these differences. Alternatively, it is possible that these two forms of victimization are more likely to result in feelings of sadness, worthlessness, loneliness, anxiety and worry due to peer rejection and verbal abuse, which would in turn lead to youth turning to video games as an escape, a means to cope, or to establish new social relationships online [75]. In contrast, as physical bullying was only associated with externalizing problems, victims of physical bullying may be more likely to react to bullying by getting angry, yelling, arguing with others, getting into fights, or skipping school, and turning to video games as a way of releasing their aggressive impulses or as a means of sensation seeking [46]. This would be in line with research identifying stronger associations between physical bullying and externalizing problems [60,61,62,63]. Overall, although these explanations require further empirical research utilizing a longitudinal design to properly test these hypotheses, the results of this study may point to different pathways to PVG severity for victims of bullying through the presence of internalizing and/or externalizing problems.

Interestingly, cyberbullying was significantly negatively associated with PVG through both internalizing problems and gaming frequency, which is in contrast to previous research identifying positive relationships between cyberbullying, gaming frequency [43,44,45,46,47] and problematic internet use [76]. It is possible that this relationship is negative because individuals who are cyberbullied could be bullied through and during their online gaming activities and as such, avoid excessive gaming as a means to avoid cyberbullying. Although research on cyberbullying and gaming is sparse, some studies have found increased rates of cyberbullying are present in online multi-player games within both adolescent and adult samples [49,77], with increased rates of cyberbullying being endorsed among those playing violent video games [45,51]. Additionally, research participants who have endured cyberbullying within online games report that it can be perceived as significantly more challenging because the perpetrators tend to be strangers. As such, anonymous cyberbullying can be identified as more severe than in-person bullying because the anonymity instigates greater feelings of helplessness within victims [78]. Overall, additional research is necessary to investigate the mediums through which cyberbullying happens (e.g., social media, forums, online gaming) while clearly establishing the directionality of these findings (i.e., are victims of cyberbullying less likely to report frequent gaming/symptoms of PVG or are those that report less frequent gaming/no symptoms of PVG less likely to by victims of cyberbullying).

For the third hypothesis, the frequency of video game play was only a significant mediator in the relationship between internalizing problems and PVG severity for verbal, indirect, and cyber bullying, with this association being negative for cyberbullying. This is in line with previous research identifying that youth victims of school bullying report increased time playing video games [43,47]. However, these results are inconsistent with previous research identifying positive relationships between time playing video games and cyberbullying victimization [43,44,46,47]. The differentiation between video game playing and PVG, in addition to the motivations towards video game playing, remains an important area of future investigation as it may yield relevant insights into differing motivations towards gaming engagement for victims of bullying (e.g., escapism, achievement, competition, social recognition, belongingness) [46]. Moreover, although the frequency of video game play was included in the present model as a mediator between externalizing and internalizing problems and PVG severity, additional longitudinal research should investigate the temporal sequencing of these variables and potential bidirectional effects.

### Limitations

The present study demonstrates a significant relationship between different types of bullying and PVG mediated through mental health problems and problem behaviors. Nevertheless, several limitations should be noted. First, as the data are cross sectional, it is not possible to assess causal relationships between the variables. Future studies should examine this relationship longitudinally in order to better evaluate the temporal nature of these relationships. Second, self-report data were utilized, which allows for potential biases in responding. Third, the survey did not include measures to identify whether some victims were also bullying others (i.e., bully-victims). Future research should investigate the differences in these different types of victimization and perpetration in order to better understand the experiences of the exhibited internalizing and externalizing problems. Finally, although a large sample size was used, the data were collected from one county in the United States (Wood County, Ohio) which included a predominantly white sample. Wood County, Ohio is located in the American Midwest and has an estimated population of 130,817, with 85.4% of households having a broadband internet subscription and a median household income of $62,390 USD [79]. As such, the present sample may not be representative of adolescents from different regions of the United States or from different countries. Additional studies should examine this relationship across multiple states and countries in order to verify the generalizability of these results.

## 5. Conclusions

The current study provides a more detailed analysis of the relationship between different types of bullying victimization and PVG severity. With the need for additional research investigating the possible mediators in the relationship between bullying victimization and PVG, this study provides an overview of the mediating role of mental health symptoms and problem behaviors while accounting for relevant variables including age, gender and the frequency of video game play. Given these findings, it appears as though externalizing and internalizing problems are of significant, but differing importance when investigating the psychological impact of different forms of bullying victimization. These results highlight a potential externalizing pathway to PVG for adolescent victims of physical, verbal and indirect bullying, and an internalizing pathway to PVG for youth victims of verbal and indirect bullying. Moreover, these results call attention to the need for a more detailed investigation of cyberbullying and understanding the online venues where youth experience these forms of victimization and how this is associated with PVG severity.

Overall, these results point to the importance of prevention and educational initiatives aimed at reducing bullying within and outside schools, with the necessary involvement of parents, guardians, and educators to monitor youth well-being and the potential mental health ramifications of bullying victimization. As the relationship between bullying victimization and PVG severity appears to be completely explained by externalizing and internalizing problems (with the exception of indirect bullying), interventions addressing youth resilience and promoting psychological and emotional well-being have the potential to be effective in reducing the negative behavioral sequalae of peer victimization. For instance, universal intervention programs targeting emotional awareness and emotion regulation can be delivered in educational settings, with the potential of having impacts at the level of primary and secondary prevention while having population-level benefits [80]. A two-pronged approach of preventing bullying victimization and promoting youth mental health is crucial to support the health and well-being of adolescents.

## Figures and Tables

**Table 1:** Participant characteristics.

Characteristic	*n*	%
Gender (Male)	3199	50.4
Age _1_	6353	14.74 (1.76)
Ethnicity (White)	5103	80.3
Grade in school		
7	1080	17.0
8	994	15.6
9	1033	16.3
10	1067	16.8
11	1100	17.3
12	887	14.0
Report bullying in the past month _2_		
Physical bullying	667	10.5
Verbal bullying	1710	26.9
Cyber bullying	825	12.9
Indirect bullying	1330	20.9
Externalizing problems _1_	5937	7.27 (7.95)
Internalizing problems _1_	5999	7.49 (10.13)
Gaming frequency _1_	6263	1.88 (1.73)
IGDS-9-SF (Problem video gaming) _1_	6085	13.08 (6.79)

Note. _1_ = Mean (standard deviation). _2_ = For physical, verbal, cyber and indirect bullying, *n* represents the number of participants endorsing bullying at least once in the past month.

**Table 2:** Pearson’s correlation coefficients between variables.

Variable	1	2	3	4	5	6	7
1. Physical bullying							
2. Verbal bullying	0.58 **						
3. Cyber bullying	0.56 **	0.57 **					
4. Indirect bullying	0.49 **	0.68 **	0.60 **				
5. Externalizing problems	0.22 **	0.32 **	0.28 **	0.32 **			
6. Internalizing problems	0.23 **	0.42 **	0.34 **	0.44 **	0.62 **		
7. Gaming frequency	0.03 *	0.04 **	0.02	0.02	0.15 **	0.09 **	
8. PVG symptoms	0.11 **	0.12 **	0.12 **	0.09 **	0.28 **	0.22 **	0.32 **

Note: * *p* < 0.05; ** *p* ≤ 0.001. PVG = problem video gaming.

**Table 3:** Indirect effects from the parallel-serial mediation model.

Indirect Effect	β (95% CI)	*p*
Physical bullying → Problem Video Gaming		
→ Externalizing problems	0.011 (0.004, 0.020)	0.009
→ Internalizing problems	0.000 (−0.009, 0.010)	0.924
→ Externalizing problems and gaming frequency	0.000 (0.000, 0.001)	0.144
→ Internalizing problems and gaming frequency	0.000 (0.000, 0.000)	0.929
Verbal bullying → Problem Video Gaming		
→ Externalizing problems	0.028 (0.018, 0.040)	0.000
→ Internalizing problems	0.065 (0.050, 0.081)	0.000
→ Externalizing problems and gaming frequency	0.001 (0.000, 0.002)	0.101
→ Internalizing problems and gaming frequency	0.002 (0.001, 0.004)	0.003
Cyber bullying → Problem Video Gaming		
→ Externalizing problems	0.006 (−0.004, 0.015)	0.245
→ Internalizing problems	−0.019 (−0.029, −0.011)	0.000
→ Externalizing problems and gaming frequency	0.000 (0.000, 0.001)	0.426
→ Internalizing problems and gaming frequency	−0.001 (−0.001, 0.000)	0.013
Indirect bullying → Problem Video Gaming		
→ Externalizing problems	0.018 (0.010, 0.030)	0.000
→ Internalizing problems	0.029 (0.019, 0.039)	0.000
→ Externalizing problems and gaming frequency	0.000 (0.000, 0.001)	0.101
→ Internalizing problems and gaming frequency	0.001 (0.000, 0.002)	0.005

## Data Availability

The data presented in this study are available on request from the corresponding author. The data are not publicly available for privacy reasons.
